# Radial Shock Wave Devices Generate Cavitation

**DOI:** 10.1371/journal.pone.0140541

**Published:** 2015-10-28

**Authors:** Nikolaus B. M. Császár, Nicholas B. Angstman, Stefan Milz, Christoph M. Sprecher, Philippe Kobel, Mohamed Farhat, John P. Furia, Christoph Schmitz

**Affiliations:** 1 Extracorporeal Shock Wave Research Unit, Department of Anatomy II, Ludwig-Maximilians-University of Munich, Munich, Germany; 2 AO Research Institute Davos, Davos, Switzerland; 3 Hydraulic Machines Laboratory, École Polytechnique Fédérale de Lausanne, Lausanne, Switzerland; 4 SUN Orthopaedic Group, Lewisburg, Pennsylvania, United States of America; University of Szeged, HUNGARY

## Abstract

**Background:**

Conflicting reports in the literature have raised the question whether radial extracorporeal shock wave therapy (rESWT) devices and vibrating massage devices have similar energy signatures and, hence, cause similar bioeffects in treated tissues.

**Methods and Findings:**

We used laser fiber optic probe hydrophone (FOPH) measurements, high-speed imaging and x-ray film analysis to compare fundamental elements of the energy signatures of two rESWT devices (Swiss DolorClast; Electro Medical Systems, Nyon, Switzerland; D-Actor 200; Storz Medical, Tägerwillen, Switzerland) and a vibrating massage device (Vibracare; G5/General Physiotherapy, Inc., Earth City, MO, USA). To assert potential bioeffects of these treatment modalities we investigated the influence of rESWT and vibrating massage devices on locomotion ability of *Caenorhabditis elegans* (*C*. *elegans*) worms.

**Results:**

FOPH measurements demonstrated that both rESWT devices generated acoustic waves with comparable pressure and energy flux density. Furthermore, both rESWT devices generated cavitation as evidenced by high-speed imaging and caused mechanical damage on the surface of x-ray film. The vibrating massage device did not show any of these characteristics. Moreover, locomotion ability of *C*. *elegans* was statistically significantly impaired after exposure to radial extracorporeal shock waves but was unaffected after exposure of worms to the vibrating massage device.

**Conclusions:**

The results of the present study indicate that both energy signature and bioeffects of rESWT devices are fundamentally different from those of vibrating massage devices.

**Clinical Relevance:**

Prior ESWT studies have shown that tissues treated with sufficient quantities of acoustic sound waves undergo cavitation build-up, mechanotransduction, and ultimately, a biological alteration that “kick-starts” the healing response. Due to their different treatment indications and contra-indications rESWT devices cannot be equated to vibrating massage devices and should be used with due caution in clinical practice.

## Introduction

**Fig 1:**
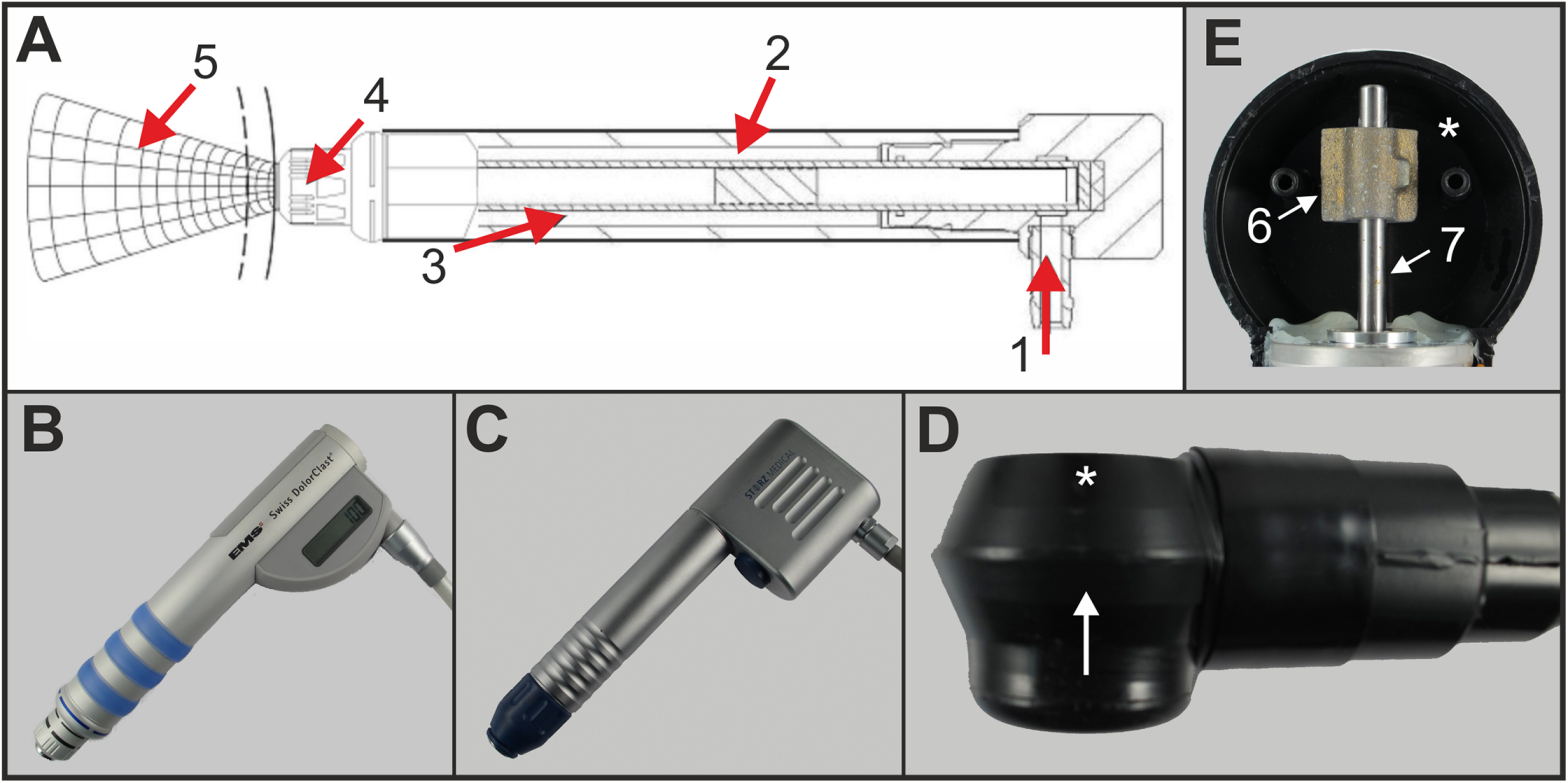
Devices investigated in the present study and their working principles. (A) Working principle of radial extracorporeal shock wave therapy (rESWT) devices. Compressed air (1) is used to fire a projectile (2) within a guiding tube (3) that strikes a metal applicator (4) placed on the patient’s skin. The projectile generates stress waves in the applicator that transmit pressure waves (5) non-invasively into tissue. Note that both the Swiss DolorClast (B) and the D-Actor 200 (C) share this construction principle. (B) “Radial” handpiece of the Swiss DolorClast (EMS) with the 15-mm applicator. (C) Handpiece of the D-Actor 200 (Storz Medical) with the 15-mm applicator. (D) Vibracare (G5/General Physiotherapy). The arrow indicates the direction of view into the chamber of the Vibracare head that was opened in (E); the asterisk indicates the backside of the chamber. (E) Working principle of the Vibracare. A flywheel mass (6) rotates around a vertical axis (7) within a chamber (asterisk).

Radial extracorporeal shock wave therapy (rESWT) is widely used in the non-invasive treatment of various diseases of the musculoskeletal system and other soft tissue disorders (see, e.g., [1–3]). Several studies addressed the molecular and cellular mechanisms of rESWT on these conditions including the mediation of cell apoptosis, enhanced angiogenesis and wound healing as well as new bone formation (see, e.g., [4,5,6]). The working principle of rESWT devices is illustrated in Fig 1A. Compressed air (or an electromagnetic field) is used to fire a projectile within a guiding tube that strikes a metal applicator placed on the patient’s skin. The projectile generates stress waves in the applicator that transmit pressure waves (radial shock waves) non-invasively into tissue.

Because radial shock waves are not real shock waves in the strict physical sense (for details see [2,7,8]) some authors called rESWT “radial pressure wave treatment (RPWT)” [4] or “radial pulse therapy (RPT)” [9]. Furthermore, conflicting reports exist in the literature as to whether at all radial extracorporeal shock waves (rESW) can generate cavitation, which refers to the rapid formation, expansion, and forceful collapse of vapor bubbles in liquids subject to rapid pressure changes [2,10,11]. Cavitation can, next to exerting therapeutic bioeffects, also produce unwanted side effects including hematomas, blood vessel rupture, and permanent injury to organs such as kidneys and lungs [11–17]. In this respect an early review about ESWT published in 2003 [18] concluded that for the only rESWT device available at that time, the Swiss DolorClast (Electro Medical Systems, Nyon, Switzerland) (Fig 1B), it was not possible to detect cavitation at all. Three years later, another group reported that this rESWT device was in fact capable of generating cavitation [7]. Since then the Swiss DolorClast has been used in many prospective, randomized controlled clinical trials that are listed in the open access Physiotherapy Evidence Database, PEDro [19] (i.e. [20–38]).

Recently a novel device, D-Actor 200 (Storz Medical, Tägerwilen, Switzerland) (Fig 1C), was introduced into the treatment for Achilles tendinopathy [39], calf strains [40] and cellulite [41]. Yet whereas one research group called this treatment modality “low-energy radial-pulsed–activated (EPAT) shockwave (sound wave)” and referred to the D-Actor 200 as a radial shock wave device [39] the other research group named treatments performed with the D-Actor 200 “Acoustic Wave Therapy (AWT)” and called the D-Actor 200 a “vibrating massage system (EPAT) that operates via compressed air to perform AWT on targeted tissue” [41]. In fact, the manufacturer of the D-Actor 200 (Storz Medical) listed several vibrating massage devices as predicate device of the D-Actor 200 in Appendix G of the 510[k] summary of the D-Actor 200 with the U.S. Food and Drug Administration (FDA). Among these vibrating massage devices is the Vibracare (G5/General Physiotherapy, Earth City [St. Louis], MO, USA) (Fig 1D). The latter device is electrically powered and causes vibrations by means of a flywheel mass that rotates around a vertical axis within a chamber (Fig 1E). In fact, with regard to design and working principle the D-Actor 200 appears very similar to the Swiss DolorClast but very different to that of vibrating massage devices such as the Vibracare. Furthermore, to our knowledge the Vibracare has so far only been studied for percussion treatment of patients with cystic fibrosis [42] and children with cerebral palsy suffering from lung infections [43], but not for treatments of diseases of the musculoskeletal system and cellulite. On the other hand, the D-Actor 200 must not be applied over air-filled tissue such as the lung.

These conflicting descriptions of the D-Actor 200 in the literature have raised the general question whether or not the energy signature of rESWT devices resembles the energy signature of vibrating massage devices. In particular, we compared the energy signature of the D-Actor 200 and the Swiss DolorClast to the energy signature of the Vibracare. This was done by applying techniques that have been established in the literature for the characterization of the energy signature of therapeutic ESW devices: (i) acoustic measurements using a laser fiber optic probe hydrophone (FOPH) [11,44]; (ii) high-speed imaging of cavitation bubbles [45,46]; and (iii) exposure of x-ray films to pressure waves [10]. Furthermore, to investigate cavitation-mediated bioeffects induced by rESWT devices and/or vibrating massage devices we analyzed cultures of the nematode worm *Caenorhabditis elegans* (*C*. *elegans*) for locomotion ability after their exposure to either treatment modality, a method that has been established recently for radial shock waves generated with the Swiss DolorClast [47].

## Methods

### Investigated devices

The following devices and applicators were investigated in the present study (see also Fig 1): (i) D-Actor 200 (Model 2007; Storz Medical) operated with the 15-mm applicator as used in [39]; (ii) Swiss DolorClast (EMS) operated with the “Radial” handpiece and the 15-mm applicator as used in many clinical trials (e.g., [21,22,25,27,29,30]); and (iii) Vibracare (Item SKU VC24B; G5/General Physiotherapy, Inc.).

The Supporting Information (S1 File, S1 and S2 Figs) contains additional data from high-speed imaging analysis of cavitation (outlined further down) generated by the D-Actor 200, Swiss DolorClast (“Radial” handpiece) and the following rESWT devices/handpieces: (i) BTL-5000 SWT Power (BTL, Prague, Czech Republic) operated with the 15-mm applicator (used in [48]); (ii) Swiss DolorClast (EMS) operated with the “EvoBlue” and “Power+” handpieces and their 15-mm applicators; and (iii) en Puls V. 2.0 (Zimmer, Neu-Ulm, Germany) with its 15-mm applicator.

### Acoustic measurements using a laser fiber optic probe hydrophone

Measurements of the pressure of the acoustic waves generated by the D-Actor 200 and the Swiss DolorClast were carried out according to IEC-61846:1998 (Ultrasonics—Pressure pulse lithotripters—Characteristics of fields) in a tank of 300 liters filled with demineralized water (conductivity approximately 5μS/cm) at the laboratories of Electro Medical Systems (Nyon, Switzerland). The inner dimensions of the tank were 960×560 mm with a height of 660 mm. The water level rose to 470 mm when the applicators were immerged at a height of 330 mm.

Measurements were performed with a laser fiber optic probe hydrophone (FOPH 2000; RP Acoustics, Leutenbach, Germany) coupled to an oscilloscope (LeCroy 9361; LeCroy, Chestnut Ridge, NY, USA) in the x-axis of the applicator. Positioning of the laser hydrophone probe was controlled with step motors, allowing a resolution of the position of 0.1 mm. Measurements were performed at various distances to the applicators (1, 5, 10 and 20 mm) and operating the D-Actor 200 and the Swiss DolorClast at various air pressures (D-Actor 200: 3 bar and [maximum] 5 bar; Swiss DolorClast: 3 bar and [maximum] 4 bar). All measurements were repeated five times and the results were averaged.

The electrical signal recorded by the oscilloscope was linked to the pressure signal (*P*) according to Eq 1:

$$P=-412MPa\times \left(1+\alpha \right)\times \frac{\Delta U}{U_{Water}-U_{B}}$$ (1)

with α the reflection factor (given at 0.07), Δ*U* the measured electrical signal, *U*_*Water*_ the reference voltage of the noise of the measurement (probe without laser), and *U*_*B*_ the reference voltage of the probe (with laser activated).

The energy flux density (*J*) is the integral of the pressure as shown in Eq 2:

$$J=\frac{1}{Z}\int_{a}^{b}P{\left(t\right)}^{2}dt$$ (2)

with *Z* the impedance of sound in water (1.5×10^6^ kg×m^-2^×s^-1^), *P(t)* the pressure as a function of time, *a* the first positive extreme of the first measured pressure peak, and *b* the second positive extreme of the first measured pressure peak.

Results were graphically represented using GraphPad Prism software (version 5; GraphPad, San Diego, CA, USA).

Because of the oscillating movements of the Vibracare head it was not possible to investigate this device with the laser hydrophone.

### High-speed imaging of cavitation bubbles

These investigations were performed at the Hydraulic Machines Laboratory of the École Polytechnique Fédérale de Lausanne (Lausanne, Switzerland). The tips of the 15-mm applicators of the D-Actor 200 and the Swiss DolorClast, mounted on their respective handpieces, as well as the tip of the Vibracare head were submerged one after another in de-ionized water contained within a custom-built transparent cubic vessel made of clear high-density polycarbonate (20 cm side length, 1 cm wall thickness). Measurements of the D-Actor 200 were performed at 3 bar and (maximum) 5 bar air pressure, and measurements of the Swiss DolorClast at 3 bar and (maximum) 4 bar air pressure. Measurements were performed at 1 Hz and 15 Hz. The Vibracare was operated at maximum energy settings (i.e. 50 cycles per second, according to the operation manual). All measurements were run in triplicates. The water was systematically degassed before each test to reduce the nuclei content. To this end, a vacuum pump connected to the vessel was operated for several minutes.

To monitor the cavitation occurrence for each device, a high-speed charge coupled device (CCD) camera (Photron Ultima APX; Photron, Tokyo, Japan) with a framing rate of 300,000 frames per second and exposure time of 1/2,700,000 seconds was used. Each captured frame comprised a total of 8.192 (64 x 128) pixels, encompassing an area of approximately 8.8 x 17.6 mm. A parallel LED background illumination (IMG Stage Line 3W LED-36Spot; Monacor International, Bremen, Germany) provided a white background, against which cavitation bubbles appear as black absorption features. Using the minimal exposure time of 370 ns, this illumination also allows the visualization of shock waves in shadowgraphy [49].

The applicators of the D-Actor 200 and the Swiss DolorClast were lowered from above into the camera frame’s top section. Camera recordings were triggered manually prior to the release of a single pulse generated by the D-Actor 200 or the Swiss DolorClast, respectively. Individual film sequences were subsequently visualized using FASTCAM viewer software (Photron, Tokyo, Japan), converted into individual images with 256 greyscales (with zero and 256 representing black and white, respectively), exported as TIF files, and then reduced for data analysis to 1,001 frames each (equivalent to film duration of 3.3 ms) to capture only those frames that showed the cavitation peak caused by a single pulse generated by the D-Actor 200 or the Swiss DolorClast, respectively, plus 500 frames before and after the cavitation peak (i.e., with the peak in between). In case of the Vibracare the head of the device was brought from the side into the camera’s field-of-view, the camera was switched on during continuous running of the device, and film sequences were reduced for data analysis to 10,000 frames.

Quantitative evaluation of the individual frames for the presence or absence of labeled pixels caused by cavitation bubbles was performed using a custom macro for Zeiss KS400 software (Carl Zeiss Vision, Eching, Germany). Each greyscale image was binarized with the threshold set to a grey level of 35, and the number of labeled pixels was determined. To account for potential artifacts in the film sequences (caused by, e.g., dirt in the water introduced during the measurements) the numbers of labeled pixels found in the first frame were subtracted from the corresponding numbers of labeled pixels found in all consecutive frames (no. 2–1001) of the corresponding film sequence.

Results were also graphically represented using Prism software (GraphPad).

### Exposure of x-ray films to pressure waves

Coleman et al. [10] reported that shock waves can blacken silver grains in x-ray films due to the mechanical impact of collapsing cavitation bubbles on the film sheets’ surface. We therefore tested the hypothesis that silver grains in x-ray films can also be blackened by exposing x-ray films to the pressure waves generated by the D-Actor 200, Swiss DolorClast and Vibracare devices. Measurements were performed at the Department of Anatomy II of the Ludwig-Maximilians University of Munich (Munich, Germany) in a dark room that was sparsely illuminated with red light. Individual x-ray films not sensitive to the red light (STRUCTURIX, D4DW, Agfa Gevaert, Mortsel, Belgium) were fixed at their outer edges within a custom-made frame in a horizontal position such that the sheet’s main surface was left unrestrained by the frame. The entire construction was then submerged in de-ionized water. The applicators of the D-Actor 200 and the Swiss DolorClast as well as the head of the Vibracare were placed underwater, exactly 3 mm above the center of an individual x-ray sheet. To this end the handpieces of the D-Actor 200 and the Swiss DolorClast were vertically mounted on a drill-stand (Wolfcraft, Kempenich, Germany), with the applicators facing downward into the water. Then a total of 10,000 pulses each were applied to the sheets’ center at maximum energy settings (i.e., 5 bar for the D-Actor 200 and 4 bar for the Swiss DolorClast) at a frequency of 1 Hz. The Vibracare was mounted in a custom-made frame with the head facing downward into the water, and was again operated at maximum energy settings (i.e. 50 cycles per second) for one hour.

The exposed films were developed with an x-ray film developing device (Protec C2; Protec, Oberstenfeld, Germany) and examined using a Zeiss Axiophot Microscope (Zeiss, Goettingen, Germany) for evidence of blackened silver grains and potential damage. Photomicrographs were taken using a 2.5x objective (Neofluar, Zeiss) and a camera (AxioCam HRc, Zeiss) attached to the microscope.

### Influence of rESWT and vibrating massage devices on *C*. *elegans* locomotion ability

Maintenance of adult wild type nematodes (N2, Bristol) obtained from the Caenorhabditis Genetics Center (Minneapolis, MN, USA), their exposure to shock waves with subsequent transfer from liquid to agar plates as well as analysis of worm locomotion data after shock wave exposure was performed at the Department of Anatomy II of the Ludwig-Maximilians University of Munich as described in detail previously [47]. Briefly, 20 adult worms were placed together with either 300 μl S-Medium [50] or 31,000 g/mol polyvinyl alcohol (PVA) (i.e. Mowiol 4–88, Karl Roth, Karlsruhe, Germany) into non-adjacent U-bottom wells (n = 5 each for S-medium and PVA) of 96-well plates (VWR, Radnor, PA, USA). With its high viscosity PVA effectively suppresses cavitation generation, thus, by exclusion criterion, enabling to attribute shock wave effects to cavitation [14,47].

**Fig 2:**
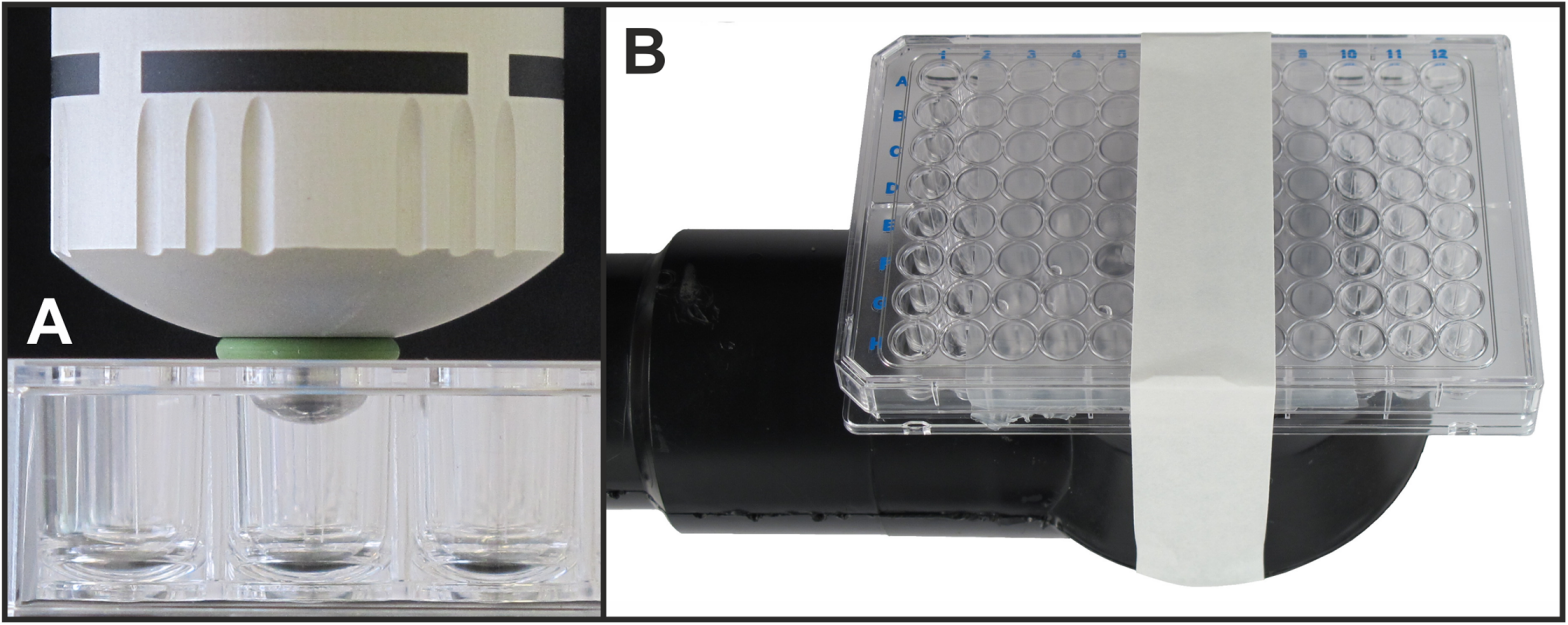
Exposure of *C*. *elegans* worms to radial shock waves and the movements of the Vibracare head. In (A) the “Radial” handpiece of the Swiss DolorClast (Electro Medical Systems) with the 6-mm applicator was lowered from above into one U-bottom well of a 96-well plate containing *C*. *elegans* worms either in S-Medium or PVA (see main text). A fluorinated rubber O-ring (green) was used to seal the U-bottom well. In (B) a 96-well plate containing *C*. *elegans* worms, sealed with parafilm and closed with its lid, was fixed with adhesive tape onto the upwards facing massaging head of the Vibracare (G5/General Physiotherapy).

In order to assess the effects of rESW on worm locomotion ability, the handpiece of the Swiss DolorClast was set vertically into a drill stand (Wolfcraft, Kempenich, Germany). Here, instead of the 15-mm applicator the handpiece of the Swiss DolorClast was equipped with its 6-mm applicator as it fits into individual wells of 96-well plates (a 6-mm applicator was not available for the D-Actor 200). The handpiece of the Swiss DolorClast was lowered from above into the well and a 5.5 x 2 mm fluorinated rubber O-ring (Vi 670/FKM 80, C. Otto Gehrckens, Pinneberg, Germany) was placed externally around the applicator to provide a tight connection with the well plate and to guard against loss of sample during rESW exposure (Fig 2A). Five hundred impulses of radial shock waves at an intensity of 2 bar (corresponding to an energy flux density of 0.08 mJ/mm^2^) and a frequency of 1 Hz were then applied to wells containing worms. To assess the effects of the Vibracare on worm locomotion ability the 96-well plates containing worms with either S-Medium or PVA (n = 5 wells each) as described were sealed with parafilm (M^®^ PM999; Pechiney Plastic Packaging, Chicago, IL, USA) to prevent sample loss. The Vibracare was again placed in the custom-made frame as described above although with the massage head facing upward. Sealed wellplates were then fixed from above on the upward facing massage head using adhesive tape (Fig 2B). The Vibracare was then operated at maximum energy settings (50 cycles per second) for 10 seconds (resulting in 500 vibrations). Control samples (n = 5 wells for each S-Medium and PVA) were treated identically except that the devices (D-Actor 200, Swiss DolorClast, Vibracare) remained switched off.

After exposure to either rESW or the movements of the head of the Vibracare worms were rapidly transferred from their liquid medium to nematode growth media (NGM) agar plates (for details see [47]). After transfer of worms NGM-agar plates were placed under a dissecting microscope (MZ75, Leica, Wetzlar, Germany; equipped with a 1.0x PlanApo objective) with an LCD light illumination set to a color temperature of 2800 K (KL 1500, Schott, Mainz, Germany). Using a 5.0 megapixel, mono digital camera (Grasshopper 2, Point Grey Research, Richmond, BC, Canada) and the video capture function of the software WormLab (Version 2.0.1, MBF Bioscience, Williston, VT) one minute long videos where then captured at 15 frames per second (FPS) with a resolution of 1280 x 960 pixels. Videos were investigated for percent of worms moving (by means of tracking worm mid-point position; i.e. x, y coordinates) and average speed of worm locomotion using Microsoft Excel 2010 (Microsoft, Redmond, WA) transformation of raw data.

Statistical analysis was performed on speed of worm locomotion data. To this end the D'Agostino and Pearson omnibus normality test of the data of all groups (separately for each group) was performed: no group passed the normality test. Kruskal-Wallis test was then performed separately for the worms in S-medium and the worms in PVA, followed each by Dunn's multiple comparison test (comparing all groups with each other). Significance was established at p < 0.05.

## Results

### Acoustic measurements using a laser fiber optic probe hydrophone

**Fig 3:**
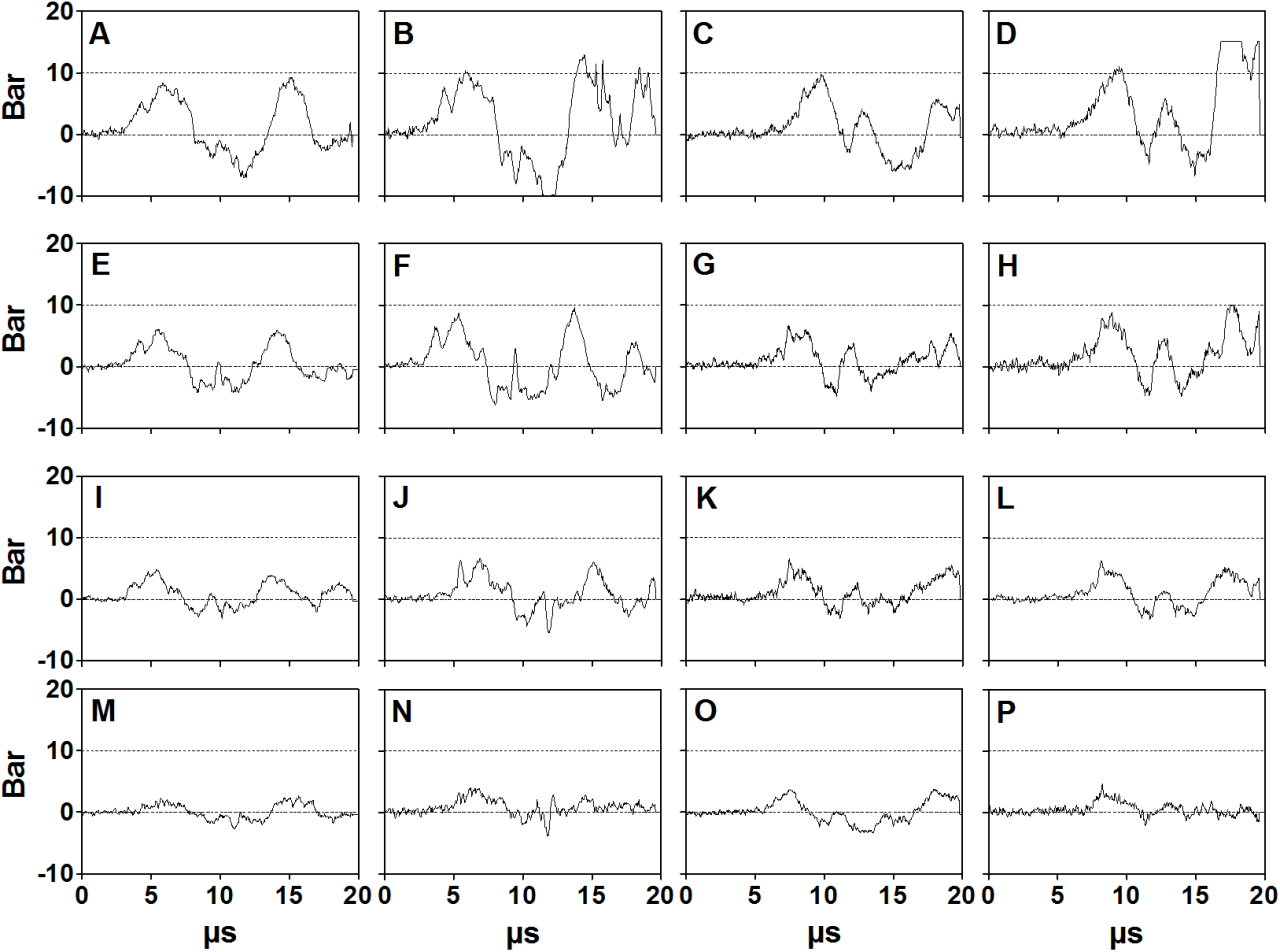
Pressure as a function of time generated by the D-Actor 200 and the Swiss DolorClast devices. The panels show the pressure as a function of time generated by the D-Actor 200 operated at 3 bar (A,E,I,M) and (maximum) 5 bar (B,F,J,N) as well as with the Swiss DolorClast operated at 3 bar (C,G,K,O) and (maximum) 4 bar (D,H,L,P). Measurements were performed five times each; the data shown here represent the measurements (one out of five repetitions) that resulted in the highest positive pressure each. Measurements were performed with a laser hydrophone at a distance of 1 mm (A-D), 5 mm (E-H), 10 mm (I-L) and 20 mm (M-P) to the applicator.

The pressure waves generated by the D-Actor 200 and the Swiss DolorClast showed very similar waveforms (Fig 3). All pressure waves were characterized by an initial peak of positive pressure (P_+_), followed by a peak of negative pressure (P_-_), and subsequent waves of positive and negative pressure. The pressure waves lasted approximately 15 μs. Table 1 summarizes P_+_, P_-_ and the energy flux densities of the pressure waves shown in Fig 3. Note that at 3 bar air pressure the pressure waves generated by the two devices had almost the same energy flux density, and operating the D-Actor 200 at (maximum) 5 bar air pressure did not result in a higher energy flux density than operating the Swiss DolorClast at (maximum) 4 bar air pressure (Table 1). For the Vibracare any potential pressure curves could not be measured due to the device’s oscillation.

**Table 1:** Peak positive pressure (P_+_), peak negative pressure (P_-_), rise time (Rt) and positive energy flux density (EFD_+_) of pressure waves generated by the D-Actor 200 and Swiss DolorClast devices at different distances to the applicator.

Device	Operating air pressure [bar]	Distance to the applicator [mm]	P_+_ [MPa]	P- [MPa]	Rt [μs]	EFD_+_ [mJ/mm^2^]
D-Actor 200	3	1	8.6	-6.7	2.6	0.10
		5	6.2	-4.5	2.7	0.04
		10	4.9	-4.2	2.1	0.02
		20	2.4	-2.8	1.3	<0.01
	5	1	10.5	-9.0	3.0	0.14
		5	8.2	-6.5	2.7	0.07
		10	6.5	-5.0	1.8	0.04
		20	3.8	-3.6	2.6	0.02
Swiss	3	1	10.1	-5.7	2.9	0.10
DolorClast		5	6.6	-4.8	1.9	0.04
		10	5.9	-3.0	1.9	0.02
		20	4.2	-2.6	2.5	0.02
	4	1	11.3	-6.0	3.4	0.14
		5	8.4	-4.8	2.5	0.06
		10	6.2	-3.2	1.8	0.03
		20	4.5	-2.0	2.5	0.01

All values represent the average of five measurements.

### High-speed imaging of cavitation bubbles

**Fig 4:**
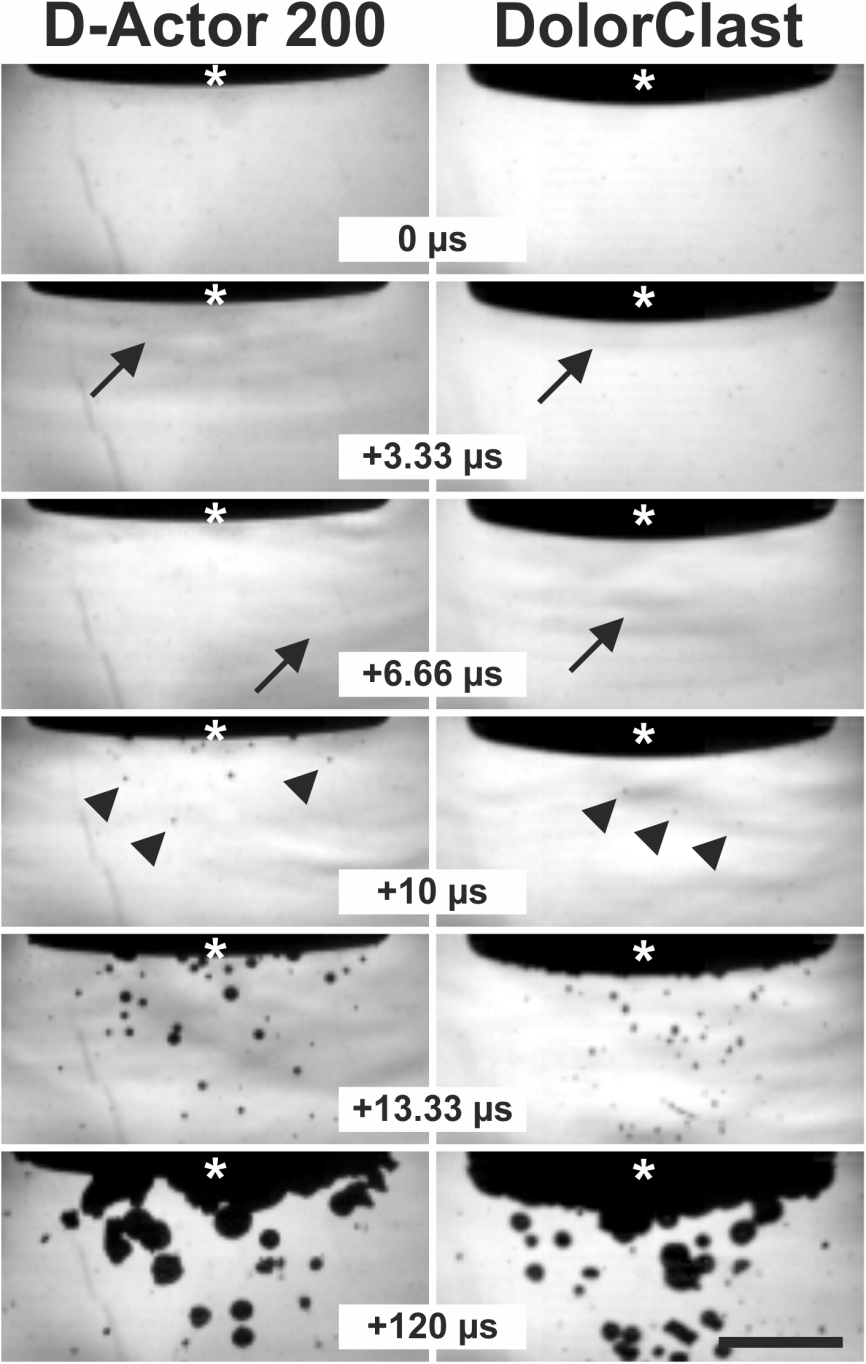
Pressure waves and cavitation bubbles generated by the D-Actor 200 and the Swiss DolorClast devices. Representative frames of the high-speed imaging experiments described in the main text, showing pressure waves (arrows) emitted from the applicators of the D-Actor 200 operated at (maximum) 5 bar air pressure (on the left) and the Swiss DolorClast operated at (maximum) 4 bar air pressure (on the right). The panels show five consecutive frames each 3.33 μs apart, plus a subsequent frame that was captured 120 μs after the first frame. Asterisks indicate the tip of the applicators lowered from above into the top section of the camera’s field-of-view. Note that the first cavitation bubbles were already detected at 10 μs after occurrence of the pressure wave (arrowheads in frames “+10 μs”). The scale bar represents 5 mm.

**Fig 5:**
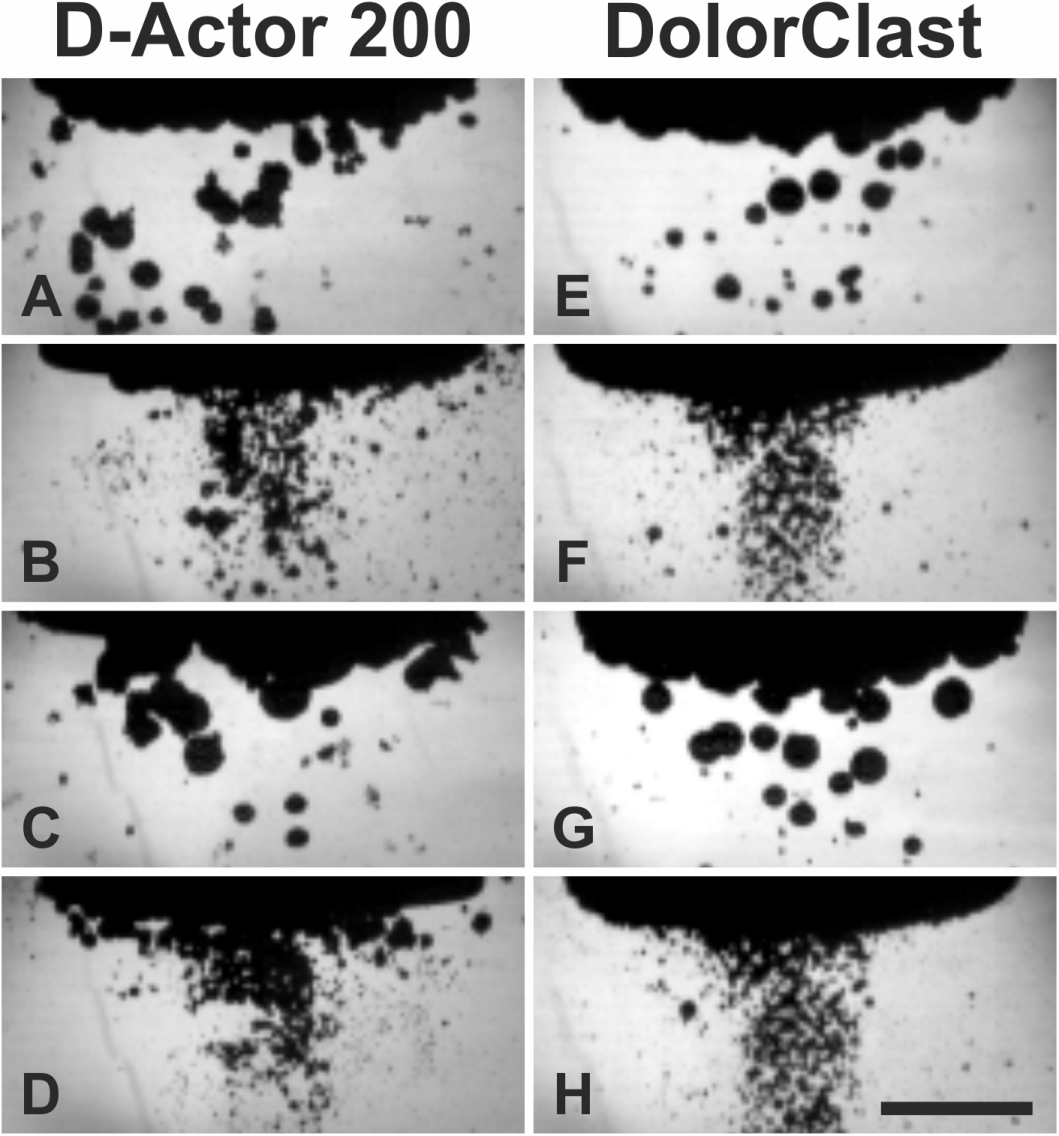
Cavitation bubbles generated by the D-Actor 200 and the Swiss DolorClast devices. The panels show the frames with the highest number of labeled pixels (from the corresponding high-speed imaging film sequences described in the main text) generated by the D-Actor 200 (A-D) operated at 3 bar and 1 Hz (A), 3 bar and 15 Hz (B), (maximum) 5 bar and 1 Hz (C), and 5 bar and 15 Hz (D), as well as with the Swiss DolorClast (E-H) operated at 3 bar and 1 Hz (E), 3 bar and 15 Hz (F), (maximum) 4 bar and 1 Hz (G), and 4 bar and 15 Hz (H). The scale bar represents 5 mm.

**Fig 6:**
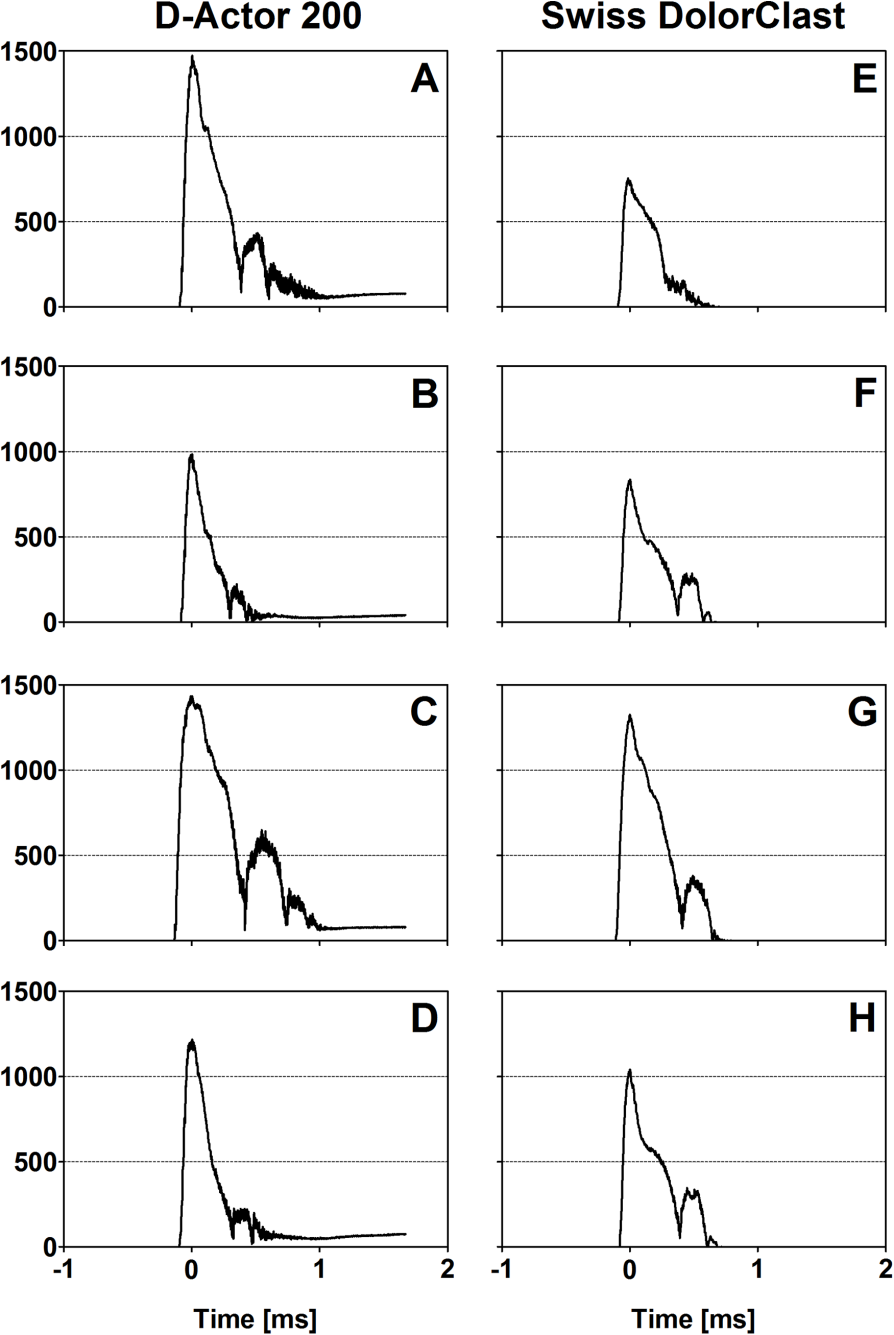
Results of the quantitative analysis of the high-speed imaging experiments. Number of detected pixels as a function of time in the high-speed imaging experiments described in the main text, obtained for the D-Actor 200 (A-D) operated at 3 bar and 1 Hz (A), 3 bar and 15 Hz (B), (maximum) 5 bar and 1 Hz (C), and 5 bar and 15 Hz (D), as well as with the Swiss DolorClast (E-H) operated at 3 bar and 1 Hz (E), 3 bar and 15 Hz (F), (maximum) 4 bar and 1 Hz (G), and 4 bar and 15 Hz (H).

High-speed imaging sequences for both the D-Actor 200 and the Swiss DolorClast revealed the build-up of cavitation bubbles as soon as 10 μs following the pressure wave front, and a cavitation maximum approximately 120 μs later (Fig 4). Both devices produced larger cavitation bubbles at 1 Hz than at 15 Hz, irrespective of the devices’ energy settings (Fig 5). Quantitative analysis of the film sequences also showed that both devices generated more cavitation at 1 Hz than at 15 Hz, with cavitation persisting for approximately 1 ms (Fig 6).

**Fig 7:**
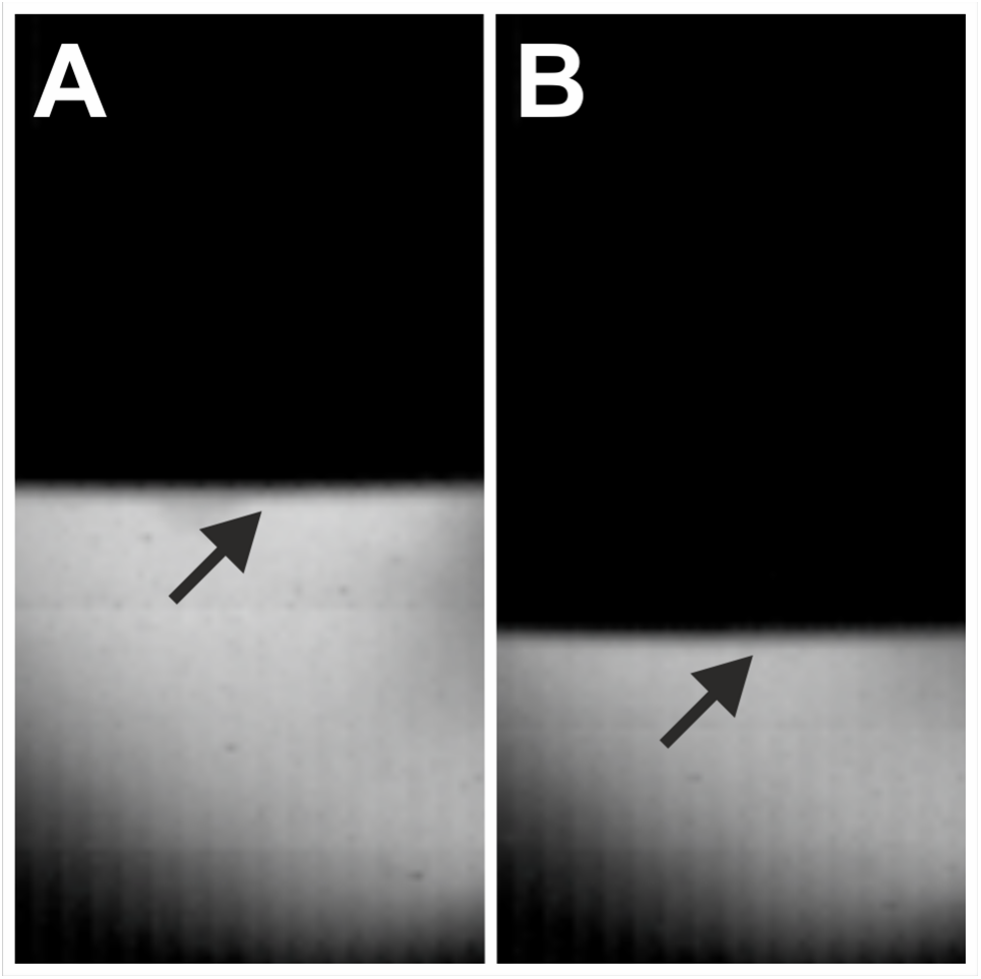
Absence of cavitation bubbles when investigating the Vibracare device with high speed imaging. The arrows point to the surface of the moving head of the device in frames of the high-speed imaging experiments, showing minimum (A) and maximum (B) deflection of the device’s head.

No cavitation bubbles were found in the high-speed imaging film sequences generated for the Vibracare (Fig 7).

### Exposure of x-ray films to pressure waves

**Fig 8:**
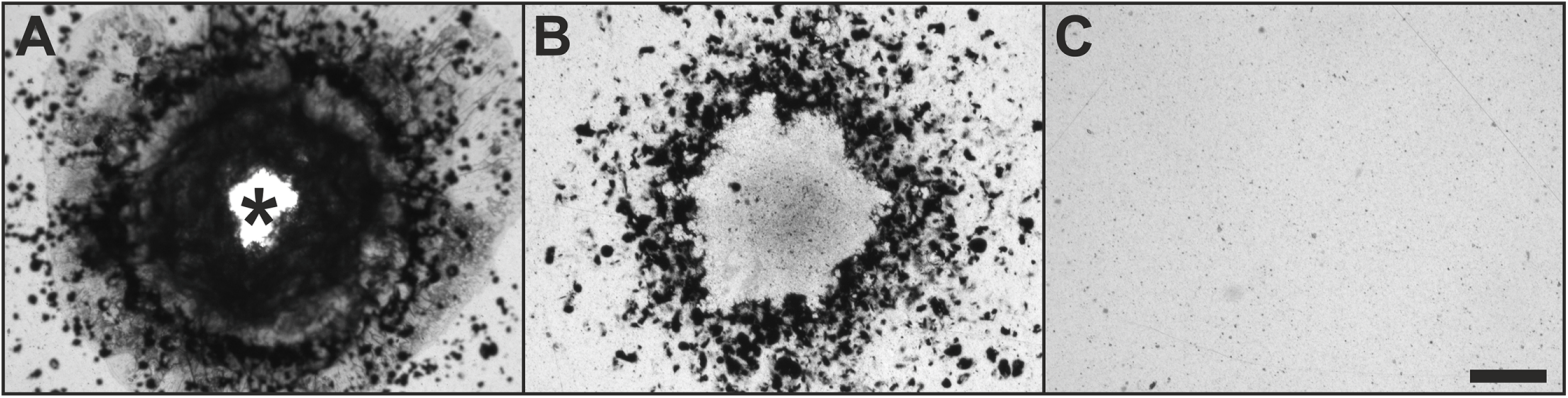
Damage of the surface of x-ray film caused by pressure waves generated by the D-Actor 200 and the Swiss DolorClast devices. The figures show the surface of x-ray film after exposure to 10,000 pressure waves generated by the D-Actor 200 (A) and the Swiss DolorClast (B) at maximum energy settings (i.e., 5 bar for the D-Actor 200 and 4 bar for the Swiss DolorClast). The asterisk in (A) indicates a hole in the x-ray film. The Vibracare device operated at maximum energy settings (50 cycles per second) had no detectable impact on x-ray film (C). The scale bars represent 500 μm.

The pressure waves generated by the D-Actor 200 and the Swiss DolorClast caused clearly discernible damage on the x-ray film’s surface (Fig 8). Damage was always characterized by a central impression surrounded by a black ring. The D-Actor 200 caused complete penetration of the x-ray film, leaving a hole within the central impression (Fig 8A). In case of the Swiss DolorClast the central impression on the x-ray film was not entirely penetrated (Fig 8B). In contrast, the Vibracare had no detectable impact on the x-ray film (Fig 8C).

### Influence of rESWT and vibrating massage devices on *C*. *elegans* locomotion ability

**Fig 9:**
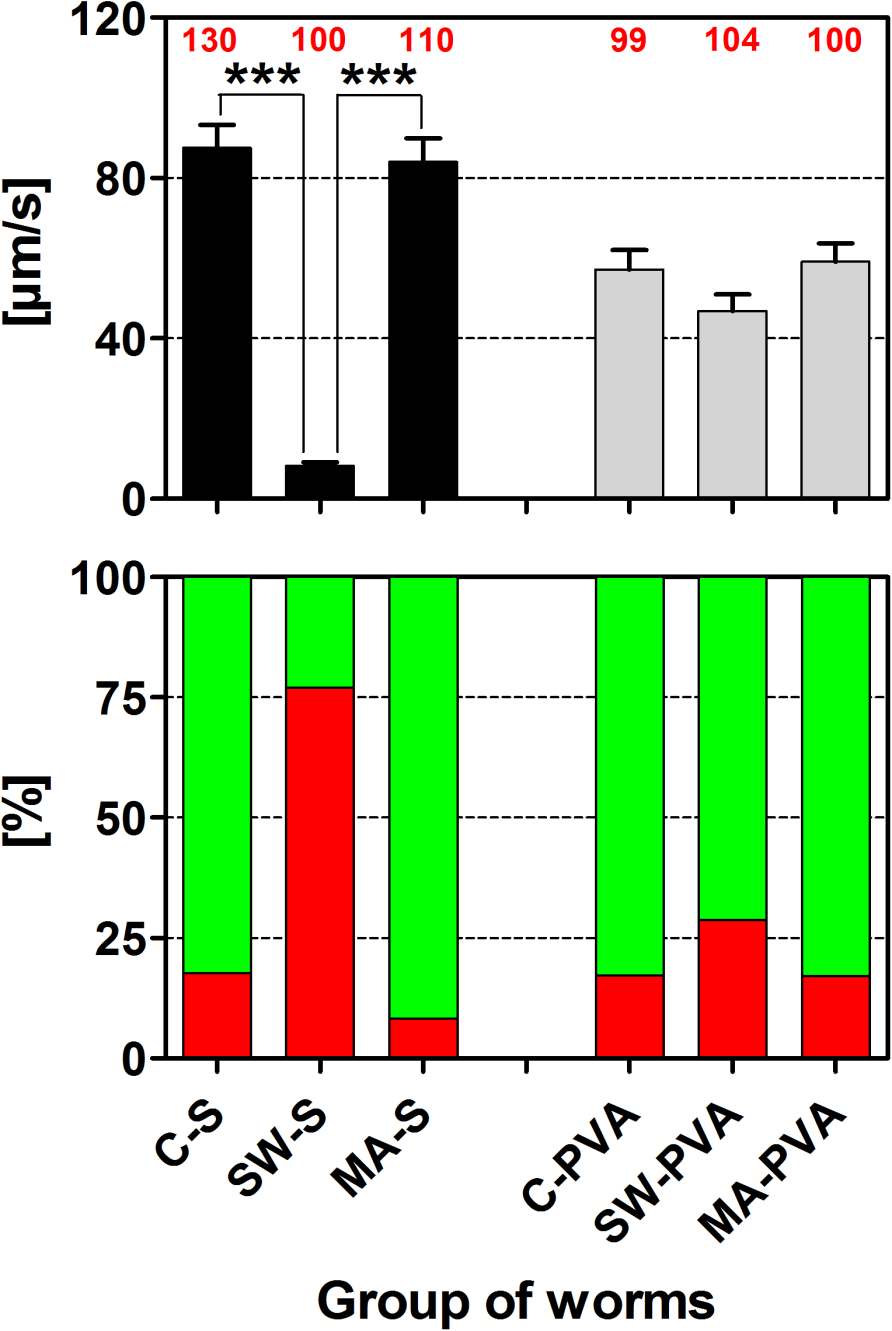
Influence of radial shock waves and the movements of the Vibracare head on *C*. *elegans* locomotion ability. The upper panel shows mean and standard error of the mean (SEM) of the speed of locomotion of the following groups of *C*. *elegans*: C-S, control worms in S-medium; SW-S, exposure of worms to 500 impulses of radial shock waves (rESW) in S-medium; MA-S, exposure of worms to the movements of the Vibracare head in S-medium; C-PVA, control worms in polyvinyl alcohol (PVA); SW-PVA, exposure of worms to 500 impulses of rESW in PVA; MA-PVA, exposure of worms to the movements of the Vibracare head in PVA. The lower panel shows the percentages of worms paralyzed (red bars) and not paralyzed (green bars) of the same groups of *C*. *elegans*. The numbers in red above the bars in the upper panel indicate the numbers of worms per group. ***, p < 0.001 (results of Dunn's multiple comparison test).

When *C*. *elegans* worms were kept in S-Medium mean speed of locomotion was statistically significantly reduced (p < 0.001) following exposure to rESW (8 ± 1 μm/s; Mean ± SEM) relative to controls (88 ± 6 μm/s) (Fig 9, top panel). Accordingly, 77% of worms exposed to rESW were rendered paralyzed compared to 18% in controls (Fig 9, bottom panel). In contrast, mean speed of movement remained virtually unchanged between worms exposed to the movements of the Vibracare head (84 ± 6 μm/s) and controls (Fig 9, top panel). Here, only 8% of worms exposed to the movements of the Vibracare head were rendered paralyzed (Fig 9, bottom panel).

When worms were kept in PVA, which effectively diminishes the build-up of cavitation bubbles, no significant differences in mean speed of movement were observed between controls (57 ± 5 μm/s) and worms exposed to either rESW (47 ± 4 μm/s) or the movements of the Vibracare head (59 ± 5 μm/s) (Fig 9, top panel). Consequently, there was no large difference in the percentage of worms paralyzed between controls (17%), and worms exposed to either rESW (29%) or the movements of the Vibracare head (17%) (Fig 9, bottom panel).

## Discussion

The main outcome of the present study was that the D-Actor 200 (Storz Medical) more closely resembles the radial extracorporeal shock wave therapy (rESWT) device Swiss DolorClast (Electro Medical Systems) but not the vibrating massage device Vibracare (G5/General Physiotherapy, Inc.), both in terms of working principle and energy signature. Both rESWT devices (D-Actor 200 and Swiss DolorClast) generated characteristic pressure waves and cavitation, and caused clearly discernible damage on x-ray films. For the Swiss DolorClast the present study confirms and expands results from earlier studies [2,3,7,8,47,51]. However, this was in sharp contrast to the vibrating massage device, whose energy signature could not be measured due to the device’s oscillation, and which neither produced cavitation bubbles nor damage on x-ray films. From its lacking potential to generate cavitation it can be deduced that the Vibracare did not generate a negative (tensile) pressure phase capable of creating cavitation [2,7]. Consequently, the vibrating massage device investigated in the present study per definition “massages” biologic tissues (respectively “move around” water in the present study) whereas rESWT devices generate acoustic pressure waves that propagate in tissues (respectively in water) and, in the process, generate cavitation. The massaging effect could be visualized in high speed imaging by the displacement of the head of the Vibracare (Fig 7) as compared to the rESWT devices’ applicator tips, which did not displace (Figs 4 and 5).

The fundamental constructional differences between rESWT devices and the vibrating massage device investigated in the present study (Fig 1) are also reflected in their different bioeffects on locomotion ability in *C*. *elegans* worms observed in the present study. We found a substantial reduction in the average speed of locomotion and an increase in the percentage of worms rendered paralyzed after their exposure in S-medium to rESW (very similar to our earlier results reported in [47]), but not to the movements of the Vibracare head. However, when worms were exposed to rESW in PVA, which diminishes cavitation due to its high viscosity, these effects were drastically reduced; thus demonstrating that cavitation was one of the biologic working mechanisms primarily responsible.

So far, two models have been established in the literature to observe cavitation-mediated bioeffects of extracorporeal shock waves, both of which utilized PVA to diminish cavitation in comparison to other fluids/media enabling cavitation. The first was an *ex vivo* model established by Schelling et al. [14], who were able to attribute the electrical stimulation of frog sciatic nerves following shock wave administration *ex vivo* unambiguously to cavitation by alternately using in their experiments Ringer’s solution and PVA. More recently, Angstman et al. [47] established a *C*. *elegans* model *in vivo*, in which worms were investigated alternately in S-medium and PVA to demonstrate cavitation-mediated bioeffects of rESW on the musculoskeletal system. Using the latter model, we were able to corroborate the finding that bioeffects of rESWT devices are indeed (at least in part) cavitation-mediated and, to demonstrate that the Vibracare device, due to its lacking cavitation generation potential, does not generate the same bioeffects than rESWT devices.

In more general terms we hypothesize that rESWT devices do not resemble vibrating massage devices, neither in terms of construction principle nor in terms of energy signature nor in terms of bioeffects induced. Hence we propose to sharply separate these two fundamentally different therapeutic systems.

It should be mentioned that for the following reasons an in-depth quantitative comparison of the cavitation output between the two rESWT devices investigated in the present study was not possible: (i) The images obtained with the high-speed camera due to their nature represent maximum intensity projections [52], which project the maximum volume elements of 3D data (i.e., the applicator’s head plus cavitation bubbles) onto the camera’s two-dimensional field-of-view. Accordingly, from all cavitation bubbles positioned along an axis perpendicular to the CCD chip the camera will only register the cavitation bubble most closely to the CCD chip. (ii) Both rESWT devices generated smaller cavitation bubbles at 15 Hz than at 1Hz. The reason for this phenomenon that has not been addressed in the technical literature, is unknown. Thus, the cavitation fields (i.e. maximum number of labeled pixels) at 15 Hz did not represent the same characteristics of the cavitation fields at 1 Hz. The images shown in Fig 5 demonstrate that it was impossible to separately count small and large cavitation bubbles.

However, the following general conclusions with high clinical relevance can be drawn: (i) Due to their cavitation generating potential, rESWT devices come with clear contraindications such as the proscribed application to target areas located above air filled tissues (e.g. lungs). The Vibracare, on the other hand, is intended for respiratory therapy applications. Thus at least one of the intended uses for the Vibracare represents a clear contraindication for rESWT devices. Characterizing the D-Actor 200 (Storz) as a vibrating massage system (as done in [41]) appears inadequate from a technical and biomedical point of view. (ii) The air pressure of rESWT devices does not predict the energy output of these devices to the patient. This is because for the devices investigated in the present study no linear relationship was found between these two parameters. For example, at 3 bar air pressure both devices generated almost exactly the same amount of energy (i.e. positive energy flux density, EFD_+_; Table 1), and yet again when operated at their highest air pressure settings although this was 5 bar in case of the D-Actor 200 and 4 bar in case of the Swiss DolorClast. (iii) Both rESWT devices investigated in the present study generated more cavitation with increasing air pressure settings. Accordingly, the intensity of rESW treatments in the clinic can be adjusted by the device settings. Again, however, no linear relationship exists between cavitation output and air pressure settings: at 3 bar the D-Actor 200 generated substantially more cavitation than the Swiss DolorClast (Fig 6) whereas both devices generated almost the same amount of cavitation when operated at their highest energy settings (i.e. 5 bar for the D-Actor 200 and 4 bar for the Swiss DolorClast). (iv) Both rESWT devices investigated in the present study produced less cavitation at 15 Hz than at 1 Hz, and this was more pronounced for the D-Actor 200 than the Swiss DolorClast. The reason for this phenomenon is unknown. This must be considered in clinical application because rESW treatments performed with these devices at high frequencies may save time but may be less effective than rESW treatments at low frequencies.

## Conclusion

This is the first study demonstrating that the potential to generate cavitation is a common feature of rESWT devices which sharply separates them from certain vibrating massage devices, the latter of which do not generate cavitation. Cavitation exerts important therapeutic bioeffects associated with shock waves, but may also cause serious negative effects on the body. Due to the non-linearity between the cavitation output and the devices’ energy settings and/or pulse frequencies, future studies should investigate the clinical effects of these observed differences among the various rESWT devices that are available today.

## Supporting Information

S1 Fig High-speed imaging of cavitation bubbles generated with radial extracorporeal shock wave devices. (TIF)

S2 Fig Results of the quantitative analysis of additional high-speed imaging experiments. (TIF)

S1 File Supporting Information. This file contains methods and results of additional experiments, as well as the corresponding references.
(DOCX)

## References

[1] Császár NB, Schmitz C. Extracorporeal shock wave therapy in musculoskeletal disorders. J Orthop Surg Res. 2013; 8: 22. 10.1186/1749-799X-8-22 PMC3726415 23895659

[2] Schmitz C, Császár NBM, Rompe JD, Chaves H, Furia JP. Treatment of chronic plantar fasciopathy with extracorporeal shock waves (review). J Orthop Surg Res. 2013; 8: 31 10.1186/1749-799X-8-31 24004715 PMC3844425

[3] Schlaudraff KU, Kiessling MC, Császár NBM, Schmitz C. Predictability of the individual clinical outcome of extracorporeal shock wave therapy for cellulite. Clin Cosmet Investig Dermatol. 2014; 7: 171–183. 10.2147/CCID.S59851 24920933 PMC4043818

[4] Contaldo C, Högger DC, Khorrami Borozadi M, Stotz M, Platz U, Forster N, et al Radial pressure waves mediate apoptosis and functional angiogenesis during wound repair in ApoE deficient mice. Microvasc Res. 2012; 84: 24–33. 10.1016/j.mvr.2012.03.006 22504489

[5] Zhao Z, Ji H, Jing R, Liu C, Wang M, Zhai L, et al Extracorporeal shock-wave therapy reduces progression of knee osteoarthritis in rabbits by reducing nitric oxide level and chondrocyte apoptosis. Arch Orthop Trauma Surg. 2012; 132: 1547–1553. 10.1007/s00402-012-1586-4 22825641

[6] Gollwitzer H, Gloeck T, Roessner M, Langer R, Horn C, Gerdesmeyer L, et al Radial extracorporeal shock wave therapy (rESWT) induces new bone formation in vivo: results of an animal study in rabbits. Ultrasound Med Biol. 2013; 39: 126–133. 10.1016/j.ultrasmedbio.2012.08.026 23122639

[7] Chitnis PV, Cleveland RO. Acoustic and cavitation fields of shock wave therapy devices. In: Clement GT, McDannold NJ, Hynynen K, editors. Therapeutic ultrasound: 5th international symposium on therapeutic ultrasound (AIP conference proceedings). Boston: AIP Conf Prot.; 2005. pp. 27–29.

[8] Cleveland RO, Chitnis PV, McClure SR. Acoustic field of a ballistic shock wave therapy device. Ultrasound Med Biol. 2007; 33: 1327–1335. 17467154 10.1016/j.ultrasmedbio.2007.02.014

[9] Speed CA. A systematic review of shockwave therapies in soft tissue conditions: focusing on the evidence. Br J Sports Med. 2014; 48: 1538–1542. 10.1136/bjsports-2012-091961 23918444

[10] Coleman AJ, Saunders JE, Crum LA, Dyson M. Acoustic cavitation generated by an extracorporeal shockwave lithotripter. Ultrasound Med Biol. 1987; 13: 69–76. 3590362 10.1016/0301-5629(87)90076-7

[11] Perez C, Chen H, Matula TJ, Karzova M, Khokhlova VA. Acoustic field characterization of the Duolith: measurements and modeling of a clinical shock wave therapy device. J Acoust Soc Am. 2013; 134: 1663–1674. 10.1121/1.4812885 23927207 PMC3745538

[12] Gerdesmeyer L, Maier M, Haake M, Schmitz C. Physical and technical principles of shock wave therapy. Orthopaede 2002; 31: 610–617. 10.1007/s00132-002-0319-8 12219657

[13] Ogden JA, Tóth-Kischkat A, Schultheiss R. Principles of shock wave therapy. Curr Orthop Rel Res. 2001; 387: 8–17. 11400898 10.1097/00003086-200106000-00003

[14] Schelling G, Delius M, Gschwender M, Grafe P, Gambihler S. Extracorporeal shock waves stimulate frog sciatic nerves indirectly via a cavitation-mediated mechanism. Biophysical J. 1994; 66: 133–140. 8130332 10.1016/S0006-3495(94)80758-1 PMC1275672

[15] Evan AP, Willis LR, McAteer JA, Bailey MR, Connors BA, Shao Y, et al Kidney damage and renal functional changes are minimized by waveform control that suppresses cavitation in shock wave lithotripsy. J Urol. 2002; 168: 1556–1562. 12352457 10.1016/S0022-5347(05)64520-X

[16] Miller DL. Overview of experimental studies of biological effects of medical ultrasound caused by gas body activation and inertial cavitation. Prog Biophys Mol Bio. 2007; 93: 314–330. 16989895 10.1016/j.pbiomolbio.2006.07.027

[17] Chen H, Brayman AA, Bailey MR, Matula TJ. Blood vessel rupture by cavitation. Urol Res. 2010; 38: 321–326. 10.1007/s00240-010-0302-5 20680255 PMC3192534

[18] McClure S, Dorfmüller C. Extracorporeal shock wave therapy: theory and equipment. Clin Tech Equine Pract. 2003; 2: 348–357.

[19] Blobaum P. Physiotherapy Evidence Database (PEDro). J Med Libr Assoc. 2006, 94: 477–478.

[20] Mehra A, Zaman T, Jenkin AI. The use of a mobile lithotripter in the treatment of tennis elbow and plantar fasciitis. Surgeon. 2003; 1: 290–292. 15570782 10.1016/s1479-666x(03)80048-3

[21] Rompe JD, Nafe B, Furia JP, Maffulli N. Eccentric loading, shock-wave treatment, or a wait-and-see policy for tendinopathy of the main body of tendo Achillis: a randomized controlled trial. Am J Sports Med. 2007; 35: 374–383. 17244902 10.1177/0363546506295940

[22] Rompe JD, Furia JP, Maffulli N. Eccentric loading compared with shock wave treatment for chronic insertional Achilles tendinopathy. A randomized, controlled trial. J Bone Joint Surg Am. 2008; 90: 52–61. 10.2106/JBJS.F.01494 18171957

[23] Marks W, Jackiewicz A, Witkowski Z, Kot J, Deja W, Lasek J. Extracorporeal shock-wave therapy (ESWT) with a new-generation pneumatic device in the treatment of heel pain. A double blind randomised controlled trial. Acta Orthop Belg. 2008; 74: 98–101. 18411608

[24] Greve JM, Grecco MV, Santos-Silva PR. Comparison of radial shockwaves and conventional physiotherapy for treating plantar fasciitis. Clinics. 2009; 64: 97–103. 19219314 10.1590/S1807-59322009000200006 PMC2666476

[25] Rompe JD, Furia JP, Maffulli N. Eccentric loading versus eccentric loading plus shock-wave treatment for midportion Achilles tendinopathy: a randomized controlled trial. Am J Sports Med. 2009a; 37: 463–470. 19088057 10.1177/0363546508326983

[26] Rompe JD, Segal NA, Cacchio A, Furia JP, Morral A, Maffulli N. Home training, local corticosteroid injection, or radial shock wave therapy for greater trochanter pain syndrome. Am J Sports Med. 2009b; 37: 1981–1990. 19439758 10.1177/0363546509334374

[27] Rompe JD, Cacchio A, Weil L Jr, Furia JP, Haist J, Reiners V, et al Plantar fascia-specific stretching versus radial shock-wave therapy as initial treatment of plantar fasciopathy. J Bone Joint Surg Am. 2010; 92: 2514–2522. 10.2106/JBJS.I.01651 21048171

[28] Chow IHW, Cheing GLY. Comparison of different energy densities of extracorporeal shock wave therapy (ESWT) for the management of chronic heel pain. Clin Rehabil. 2007; 21: 131–141. 17264107 10.1177/0269215506069244

[29] Gerdesmeyer L, Frey C, Vester J, Maier M, Weil L Jr, Weil L Sr, et al Radial extracorporeal shock wave therapy is safe and effective in the treatment of chronic recalcitrant plantar fasciitis: results of a confirmatory randomized placebo-controlled multicenter study. Am J Sports Med. 2008; 36: 2100–2109. 10.1177/0363546508324176 18832341

[30] Ibrahim MI, Donatelli RA, Schmitz C, Hellman MA, Buxbaum F. Chronic plantar fasciitis treated with two sessions of radial extracorporeal shock wave therapy. Foot Ankle Int. 2010; 31: 391–397. 10.3113/FAI.2010.0391 20460065

[31] Shaheen AAM. Comparison of three different treatment protocols of low-energy radial extracorporeal shock wave therapy for management of chronic plantar fasciitis. Ind J Physiother Occup Ther. 2010; 4: 8–12.

[32] Cacchio A, Rompe JD, Furia JP, Susi P, Santilli V, De Paulis F. Shockwave therapy for the treatment of chronic proximal hamstring tendinopathy in professional athletes. Am J Sports Med. 2011;39: 146–153. 10.1177/0363546510379324 20855554

[33] Engebretsen K, Grotle M, Bautz-Holter E, Ekeberg OM, Juel NG, Brox JI. Supervised exercises compared with radial extracorporeal shock-wave therapy for subacromial shoulder pain: 1-year results of a single-blind randomized controlled trial. Phys Ther. 2011; 91: 37–47. 10.2522/ptj.20090338 21088117

[34] Grecco MV, Brech GC, Greve JM. One-year treatment follow-up of plantar fasciitis: radial shockwaves vs. conventional physiotherapy. Clinics. 2013; 68: 1089–1095. 10.6061/clinics/2013(08)05 24037003 PMC3752632

[35] Vidal X, Morral A, Costa L, Tura M. Radial extracorporeal shock wave therapy (rESWT) in the treatment of spasticity in cerebral palsy: A randomized, placebo-controlled clinical trial. NeuroRehabilitation. 2011; 29: 413–419. 10.3233/NRE-2011-0720 22207070

[36] Liu S, Zhai L, Shi Z, Jing R, Zhao B, Xing G. Radial extracorporeal pressure pulse therapy for the primary long bicipital tenosynovitis a prospective randomized controlled study. Ultrasound Med Biol. 2012; 38: 727–735. 10.1016/j.ultrasmedbio.2012.01.024 22425375

[37] Lee SS, Kang S, Park NK, Lee CW, Song HS, Sohn MK, et al Effectiveness of initial extracorporeal shock wave therapy on the newly diagnosed lateral or medial epicondylitis. Ann Rehabil Med. 2012; 36: 681–687. 10.5535/arm.2012.36.5.681 23185733 PMC3503944

[38] Kolk A, Yang KG, Tamminga R, van der Hoeven H. Radial extracorporeal shock-wave therapy in patients with chronic rotator cuff tendinitis: a prospective randomised double-blind placebo-controlled multicentre trial. Bone Joint J. 2013; 95: 1521–1526. 10.1302/0301-620X.95B11.31879 24151273

[39] Saxena A, Ramdath S Jr, O’Halloran P, Gerdesmeyer L, Gollwitzer H. Extra-corporeal pulsed-activated Therapy (“EPAT” Sound Wave) for Achilles tendinopathy: a prospective study. J Foot Ankle Surg. 2011; 50: 315–319. 10.1053/j.jfas.2011.01.003 21406328

[40] Saxena A, St. Lois M, Fournier M. Vibration and pressure wave therapy for calf strains: a proposed treatment. Muscles, Ligaments and Tendons Journal 2013; 3: 60–62. 10.11138/mltj/2013.3.2.060 23888287 PMC3711703

[41] Russe-Wilflingseder K, Russe E, Vester JC, Haller G, Novak P, Krotz A. Placebo controlled, prospectively randomized, double-blinded study for the investigation of the effectiveness and safety of the acoustic wave therapy (AWT(®)) for cellulite treatment. J Cosmet Laser Ther. 2013; 15: 155–162. 10.3109/14764172.2012.759235 23688206

[42] Bauer ML, McDougal J, Schoumacher RA. Comparison of manual and mechanical chest percussion in hospitalized patients with cystic fibrosis. J Pediatr. 1994; 124: 250–254. 8301432 10.1016/s0022-3476(94)70313-2

[43] He YL, Liao DL, Kang HY, Ke CF, Chen YL, Liu SF, et al Comparison of mechanical insufflation-exsufflation and percussors in the treatment of lung infections for children with cerebral palsy. J Pediatr Resp Dis. 2013; 9: 40–47.

[44] Smith N, Sankin GN, Simmons WN, Nanke R, Fehre J, Zhong P. A comparison of light spot hydrophone and fiber optic probe hydrophone for lithotripter field characterization. Rev Sci Instrum. 2012; 83: 014301 10.1063/1.3678638 22299970 PMC3281968

[45] Pishchalnikov YA, Williams JC, McAteer JA. Bubble proliferation in the cavitation field of a shock wave lithotripter. J Acoust Soc Am. 2011; 130: EL87–93. 10.1121/1.3609920 21877776 PMC3195892

[46] Zhou Y, Yang K, Cui J, Ye JY, Deng CX. Controlled permeation of cell membrane by single bubble acoustic cavitation. J Control Release 2012; 157: 103–111. 10.1016/j.jconrel.2011.09.068 21945682 PMC3258473

[47] Angstman NB, Kiessling MC, Frank HG, Schmitz C. High interindividual variability in dose-dependent reduction in speed of movement after exposing C. elegans to shock waves. Front Behav Neurosci. 2015; 9: 12 10.3389/fnbeh.2015.00012 25705183 PMC4319468

[48] Gonkova MI, Ilieva EM, Ferriero G, Chavdarov I. Effect of radial shock wave therapy on muscle spasticity in children with cerebral palsy. Int J Rehabil Res. 2013; 36: 284–290. 10.1097/MRR.0b013e328360e51d 23603803

[49] Obreschkow D, Tinguley M, Dorsaz N, Kobel Ph, De Bosset A, Farhat M. The quest for the most spherical bubble. Experiments in Fluids 2013; 54: 1503 10.1007/s00348-013-1503-9

[50] Sulston JE, Brenner S. The DNA of *Caenorhabditis elegans* . Genetics. 1974; 77: 95–104. 4858229 10.1093/genetics/77.1.95 PMC1213121

[51] Kiessling MC, Milz S, Frank HG, Korbel R, Schmitz C. Radial extracorporeal shock wave treatment harms developing chicken embryos. Sci Rep. 2015; 5: 8281 10.1038/srep08281 25655309 PMC4319177

[52] Wallis JW, Miller TR, Lerner CA, Kleerup EC. Three-dimensional display in nuclear medicine. IEEE Trans Med Imaging 1989; 8: 297–303. 18230529 10.1109/42.41482

