# Supplementary figures and images for: Radial Shock Wave Devices Generate Cavitation
Source: PLoS One. 2015 Oct 28;10(10):e0140541. doi: 10.1371/journal.pone.0140541 (PMC4625004; doi:10.1371/journal.pone.0140541)

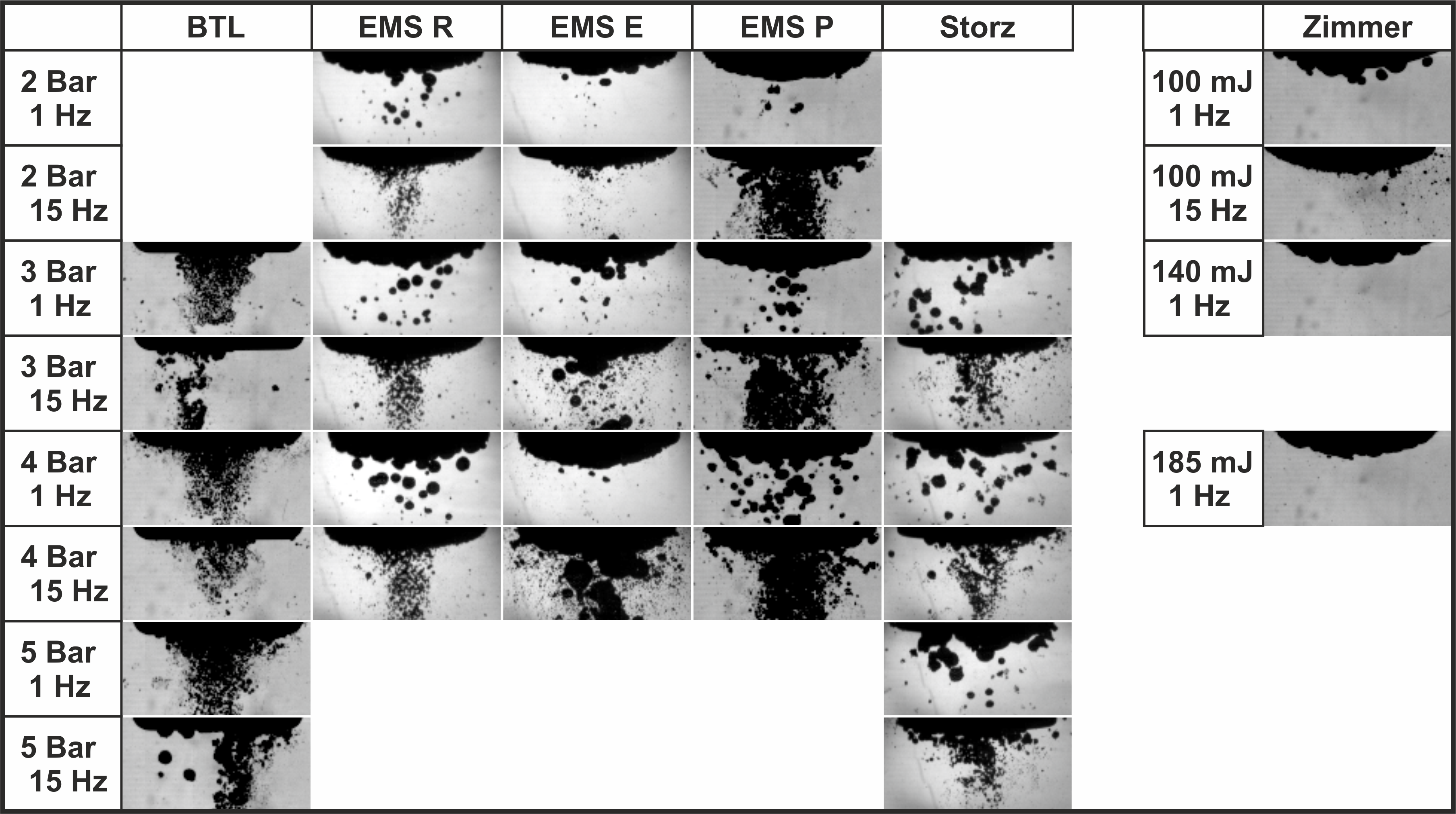

Supplement: S1 Fig — (TIF) [file pone.0140541.s001.tif]

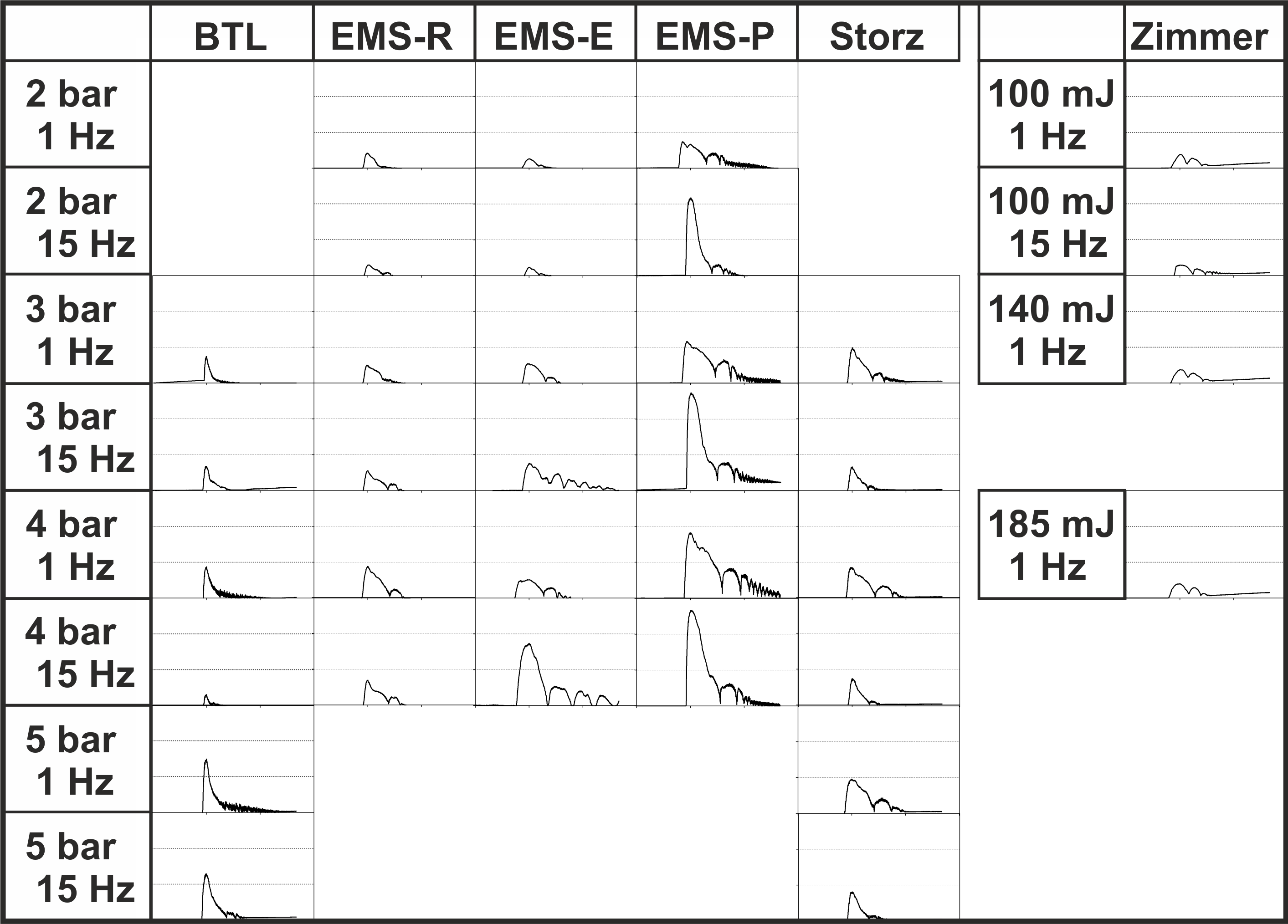

Supplement: S2 Fig — (TIF) [file pone.0140541.s002.tif]
