# Supplementary material for: Radial Shock Wave Devices Generate Cavitation
Source: PLoS One. 2015 Oct 28;10(10):e0140541. doi: 10.1371/journal.pone.0140541 (PMC4625004; doi:10.1371/journal.pone.0140541)
Supplement: S1 File — This file contains methods and results of additional experiments, as well as the corresponding references. (DOCX) [file pone.0140541.s003.docx]

**Radial shock wave devices generate cavitation**

Nikolaus B.M. Császár, Nicholas B. Angstman, Stefan Milz, Christoph M. Sprecher, Philippe Kobel, Mohamed Farhat, John P. Furia, and Christoph Schmitz

**Supporting Information**

**Methods**

The following rESWT devices (in alphabetical order of the manufacturers’ names) were investigated in the present study: (i) BTL-5000 SWT Power (BTL, Prague, Czech Republic); (ii) Swiss DolorClast (Electro Medical Systems, Nyon, Switzerland); (iii) D-Actor 200 (Storz Medical; Tägerwilen, Switzerland); and (iv) en Puls V. 2.0 (Zimmer, Neu-Ulm, Germany).

Radial ESWT devices operate on a ballistic principle by accelerating a bullet within a guiding tube to strike an applicator which eventually transforms the kinetic energy into a radially expanding pressure wave. In the devices by BTL, EMS and Storz this is achieved pneumatically whereas in the Zimmer device the ballistic principle is realized by electromagnetic means.

All four devices were operated with their respective handpieces and 15-mm applicators as used in many clinical trials (see, e.g., [1-3] for studies performed with the devices by BTL, EMS and Storz; to our knowledge no study using the Zimmer device has been published in the peer-reviewed literature). The EMS device came along with three different handpieces, named “Radial”, “EvoBlue” and “Power+”. These handpieces were evaluated separately.

The pneumatic devices were operated at different air pressure settings (BTL and Storz: 3 bar, 4 bar and [maximum] 5 bar; EMS: 2 bar, 3 bar and [maximum] 4 bar). The electromagnetic Zimmer device was operated at 100 mJ, 140 mJ and (maximum) 185 mJ. All devices were investigated at 1 Hz and at 15 Hz in the present study.

**Results**

All investigated rESWT devices generated cavitation bubbles (S1 Fig), which were constantly smaller at 15 Hz than at 1 Hz. Quantitative analysis of the film sequences showed that cavitation lasted for approximately 1 to 2 ms (S2 Fig). For the devices from BTL, EMS and Storz, the maximum number of labeled pixels (i.e. cavitation) increased with increasing air pressure (in case of the Storz device only at 15 Hz) (S2 Fig). Furthermore, the devices from BTL and Storz as well as the radial handpiece of the EMS device generated a lower maximum number of labeled pixels at 15 Hz than at 1 Hz, whereas the EvoBlue and the Power+ handpiece of the EMS device generated higher maximum numbers of detected pixels at 15 Hz than at 1 Hz. For the Zimmer device no differences between 1 Hz and 15 Hz were found at 100 mJ. At 140 mJ and 185 mJ this device cannot be operated at 15 Hz.

Therefore, the air pressure of rESWT devices does not predict the energy output of these devices to the patient. Rather the same device can, at the same air pressure, generate substantially different energy outputs to the patient depending on the handpiece technology used (as found in the present study for the EMS device).

**S1 Fig.** **High-speed imaging of cavitation bubbles generated with radial extracorporeal shock wave devices.** Each panel shows the frame with the highest number of detected pixels from the corresponding high-speed imaging film sequences described in the main text. BTL, BTL-5000 SWT Power device

(BTL, Prague, Czech Republic); EMS-R, Swiss DolorClast device (Electro Medical Systems, Nyon,

Switzerland), operated with the “radial” handpiece; EMS-E, Swiss DolorClast device, operated with the

“EvoBlue” handpiece; EMS-P, Swiss DolorClast device, operated with the “Power+” handpiece; Storz,

D-Actor 200 device (Storz Medical; Tägerwilen, Switzerland); Zimmer, en Puls V. 2.0 device (Zimmer,

Neu-Ulm, Germany).

**S2 Fig. Results of the quantitative analysis of the high-speed imaging experiments**. Number of labeld pixels (caused by cavitation) as a function of time in the high-speed imaging experiments described in the main text. In each panel the X-axis represents 3 ms, and the Y-axis the number of detected pixels (ranging from zero to 4500). BTL, BTL-5000 SWT Power device (BTL, Prague, Czech Republic); EMS-R, Swiss DolorClast device (Electro Medical Systems, Nyon, Switzerland), operated with the “radial” handpiece; EMS-E, Swiss DolorClast device, operated with the “EvoBlue” handpiece; EMS-P, Swiss DolorClast device, operated with the “Power+” handpiece; Storz, D-Actor 200 device (Storz 287 Medical; Tägerwilen, Switzerland); Zimmer, en Puls V. 2.0 device (Zimmer, Neu-Ulm, Germany).

**References**

1. Gerdesmeyer L, Frey C, Vester J, Maier M, Weil L Jr., Weil L Sr., et al. Radial extracorporeal shock wave therapy is safe and effective in the treatment of chronic recalcitrant plantar fasciitis: results of a confirmatory randomized placebo-controlled multicenter study. Am J Sports Med. 2008; 36: 2100–2109. PMID: 18832341
2. Saxena A, Ramdath S Jr., O’Halloran P, Gerdesmeyer L, Gollwitzer H. Extra-corporeal pulsed-activated Therapy (“EPAT” Sound Wave) for Achilles tendinopathy: a prospective study. J Foot Ankle Surg. 2011; 50: 315–319. PMID: 21406328
3. Russe-Wilflingseder K, Russe E, Vester JC, Haller G, Novak P, Krotz A. Placebo controlled, prospectively randomized, double-blinded study for the investigation of the effectiveness and safety of the acoustic wave therapy (AWT(®)) for cellulite treatment. J Cosmet Laser Ther. 2013; 15: 155–162.
